# Vitamin D deficiency is associated with worse neurological outcomes in moderate and severe traumatic brain injury: A prospective observational cohort study

**DOI:** 10.1097/MD.0000000000045202

**Published:** 2025-10-17

**Authors:** Mehdi Mahmoodkhani, Arvin Rahimi, Arvin Naeimi, Sepehr Aghajanian, Anita Naeimi

**Affiliations:** a Department of Neurosurgery, Isfahan University of Medical Sciences, Isfahan, Iran; b Student Research Committee, School of Medicine, Guilan University of Medical Sciences, Rasht, Iran; c Neuroscience Research Center, Iran University of Medical Sciences, Tehran, Iran; d Faculty of Medicine, Goethe University Frankfurt, Frankfurt am Main, Hesse, Germany.

**Keywords:** 25-hydroxyvitamin D, Glasgow Outcome Scale, GOSE, TBI, traumatic brain injuries, vitamin D, vitamin D deficiency

## Abstract

Vitamin D deficiency is correlated with neuroinflammation and neurocognitive defects. The present study investigated the relationship between the serum level of vitamin D and intensive care unit (ICU) length of stay, nosocomial infection, Acute Physiology and Chronic Health Evaluation II (APACHE II) score during hospitalization, and Glasgow Outcome Scale-Extended (GOSE) at 3 months in patients with moderate and severe traumatic brain injury (TBI). This prospective observational cohort study included 300 moderate-severe TBI patients admitted between May 2021 and May 2022. Demographic information and clinical characteristics of patients were collected prospectively. Serum vitamin D level (25-hydroxyvitamin D) was measured on admission and repeated 7 days after admission, as well as at the 3-month follow-up visit after admission. Vitamin D status was dichotomized into deficient (<20 ng/mL) and sufficient (≥20 ng/mL) groups. During a 90-day clinical follow-up, the GOSE, Glasgow Coma Scale, and mortality were assessed. The primary outcome was GOSE at 3 months after admission, and secondary outcomes were ICU length of stay, nosocomial infection, and APACHE II scores. Three hundred patients with a mean age of 34.98 ± 14.75 years were included. There were 222 (74%) patients with moderate TBI and 78 (26%) with severe TBI. In patients with moderate TBI, the 3-month Glasgow Coma Scale score significantly differed between vitamin D groups (*P* < .001). There was no difference in 3-month mortality between the groups. Baseline vitamin D measurements were not associated with ICU length of stay (*P* = .828), nosocomial infection (*P* = .077), and APACHE II score (*P* = .219). Vitamin D deficiency (<20 ng/mL) throughout the measurements was associated with lower GOSE scores (proportional odds ratio = 0.62, confidence interval: 0.40–0.93, *P* = .022). The results of this study suggest that vitamin D deficiency is associated with poorer neurological outcomes, as assessed by the GOSE, at 3 months. The potential role of vitamin D status as a prognostic marker and the effect of its supplementation in TBI patients should be investigated in future large-scale randomized controlled studies.

## 1. Introduction

Vitamin D is an important prohormone that regulates calcium (Ca) homeostasis. Vitamin D plays a crucial role in maintaining the function of the skeletal, cardiovascular, and central nervous systems and has a preventive effect against certain cancers.^[1]^ Vitamin D deficiency is among the most common health-related issues worldwide. Recent studies have shown that even sun-exposed countries like Saudi Arabia, the United Arab Emirates, and Qatar suffer significant vitamin D deficiency.^[2]^ In the general population, vitamin D deficiency ranges from 20% to 80%, and in severely injured patients, the prevalence of deficiency is even higher.^[3]^ To date, numerous studies have shown that 25OHD deficiency is closely correlated with various diseases, including bone metabolic disorders, cancers, cardiovascular diseases, diabetes, neuropsychiatric disorders, and autoimmune diseases.^[4]^ Additionally, medical interventions such as surgery, immobilization, fluid replacement, serum plasma exchange, and hemodialysis can also reduce serum vitamin D levels.^[5]^ In this regard, vitamin D deficiency has been demonstrated to be associated with poor clinical outcomes such as nosocomial infections, sepsis, prolonged hospitalization, and mortality or morbidity.^[6–8]^ Additionally, several studies have found an inverse relationship between vitamin D levels and markers of inflammation, such as pro-inflammatory cytokines and chemokines, suggesting that inadequate vitamin D levels might contribute to the inflammatory response.^[9]^ Hence, some authors have recently suggested that vitamin D supplementation during the acute phase of injury may reduce secondary damage, as vitamin D maintains Ca homeostasis, regulates proliferative and apoptotic activity, and plays an immunomodulatory role.^[10]^ Given the growing role of vitamin D in improving disease outcomes, a new body of research is focusing on the effects of vitamin D on lesser-known consequences.

Traumatic brain injury (TBI) is one of the leading causes of death and disability in the United States. Head trauma affects 1.5 million Americans each year, costing $56 billion annually.^[9]^ Vitamin D plays a multifaceted role in neuroprotection.^[11]^ Vitamin D supports neural protection within the central nervous system through antioxidant and detoxification mechanisms, as well as enhanced nerve conduction.^[10]^ Moreover, research suggests that vitamin D improves central nervous system function by reducing the inflammatory response after TBI, thereby preventing nerve damage and death, and lessening the inflammatory response. On the other hand, deficiencies in vitamin D increase inflammation even before an injury occurs, priming the immune system to respond more strongly after a TBI.^[12]^ In this regard, it has also been shown that there is a greater decrease in serum vitamin D levels in critically ill patients during intensive care unit (ICU) hospitalization.^[13–15]^ However, studies on the effects of vitamin D on patients with TBI are limited and require further investigation. According to this point of view, the present study examines the effect of vitamin D status on the neurological outcomes of patients with moderate and severe TBI.

## 2. Methods

### 2.1. Study design and participants

This prospective observational cohort study included all patients with moderate and severe TBI admitted to Alzahra and Kashani medical centers, affiliated hospitals of Isfahan University of Medical Sciences, from May 2021 to May 2022. A neurosurgeon initially examined all patients, and those who were diagnosed with moderate (Glasgow Coma Scale [GCS] 9–12) and severe (GCS 3–8) TBI based on the GCS were included for further evaluation. To maximize sample size and capture the breadth of clinical presentations, participants were recruited using an availability-based, consecutive sampling approach during routine visits to the aforementioned centers. The exclusion criteria were as follows: a history of taking medications that affect the metabolism, absorption, or excretion of vitamin D, including alpha blockers and Angiotensin II Receptor Blockers; use of vitamin D supplements within the last 6 months; a history of comorbidities, including cardiovascular disease (International Classification of Diseases, 10th Revision, Clinical Modification [ICD-10-CM I10-I52]), pulmonary disease (ICD-10-CM J00-J99), renal disease (ICD-10-CM N00-N29), liver disease (ICD-10-CM K70-K77), autoimmune disease (ICD-10-CM M30-M36), diseases of the digestive system (ICD-10-CM K20-K93), diseases of the nervous system (ICD-10-CM G00-G99), metabolic disorders (ICD-10-CM E70-E90), and primary hyperparathyroidism (ICD-10-CM E21); a history of bariatric surgery; a history of substance use (ICD-10-CM F10-F19); a history of alcohol use (ICD-10-CM Z72.1) recently or during the study period; a diagnosis of mild TBI on admission; the presence of hypercalcemia, defined as a total Ca level >10.6 mg/dL or ionized serum Ca exceeding 5.6 mg/dL^[16]^; lack of consent to participate in the study or failure to attend the 3-month follow-up visit. Written informed consent was obtained from the patient or next of kin when applicable. The study was approved by the ethics committee of the Isfahan University of Medical Sciences (IR.MUI.MED.REC.1400.059) in accordance with the World Medical Association’s code of ethics (Declaration of Helsinki, revised in Brazil 2013).

### 2.2. Data collection

**Figure 1.**
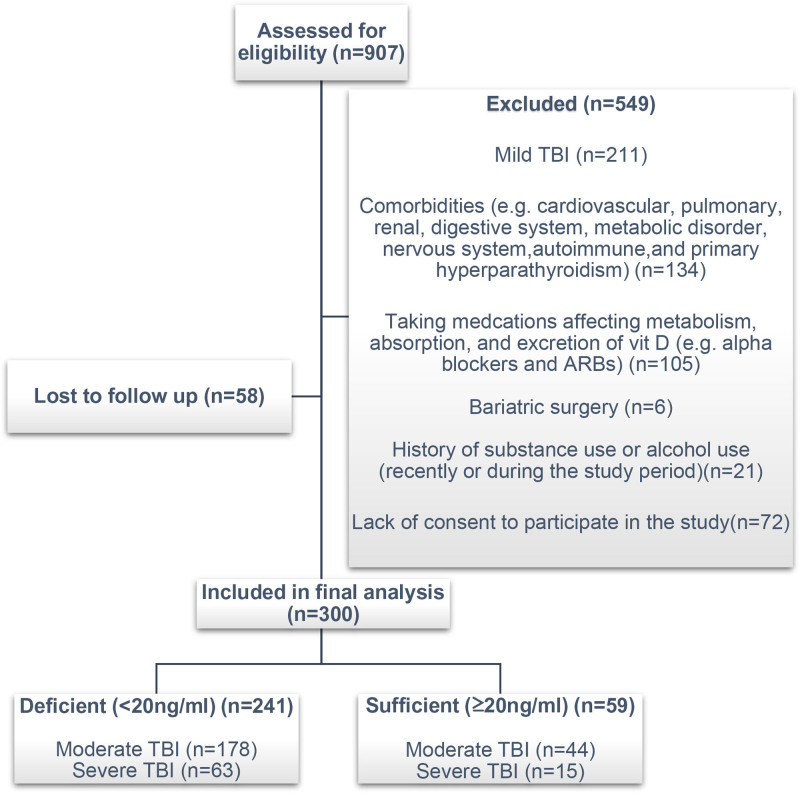
Flowchart of the study.

A total of 907 moderate and severe TBI patients were admitted during 1 year. The medical records of patients were prospectively investigated according to the inclusion criteria. Demographic information and clinical characteristics of patients, including age, gender, history of comorbidities (diabetes mellitus and hypertension), smoking, body mass index (BMI), GCS on admission, mechanism of trauma, serum levels of vitamin D, and presence of shock on admission (systolic blood pressure < 90 mm Hg or heart rate > 120/min), ICU admission, ICU length of stay, presence of nosocomial infection,^[17]^ and measures of severity including the GCS score,^[18]^ Injury Severity Score,^[19]^ Acute Physiology and Chronic Health Evaluation (APACHE) II score,^[20]^ and Simplified Acute Physiology Score III^[21]^ were collected. All variables required for analysis were complete. There were no missing data in the final dataset. According to their vitamin D levels, patients were categorized into 2 groups: deficient (<20 ng/mL [50 nmol/L]) and sufficient (greater than or equal to 20 ng/ mL [50 nmol/L]).^[22]^ The flow chart of the study is presented in Figure 1.

### 2.3. Serum level of vitamin D

The serum levels of vitamin D were determined by measuring 25-hydroxyvitamin D (25(OH)D) using liquid chromatography with mass spectrometry within the first 24 hours, 7 days, and 3 months after admission. Total serum 25(OH)D concentrations below 20 ng/mL (50 nmol/L) were considered deficient.^[22,23]^ In the case of a vitamin D deficiency, in accordance with the standard of care at our institution, a treatment regimen comprising a single oral dose of 120,000 International Units was administered within the first week of the study to individuals identified as having a vitamin D deficiency from the first measurement time point.^[12]^

### 2.4. Outcome measures

The Glasgow Outcome Scale-Extended (GOSE), GCS, and mortality were evaluated 90 days after admission in clinical follow-up by a neurosurgeon. GOSE is widely used in assessing long‐term global outcomes following a TBI. It consists of 8 outcomes, including upper and lower good recovery, upper and lower moderate disability, upper and lower severe disability, vegetative state, and death.^[24]^ In addition, ICU length of stay, nosocomial infection, and Acute Physiology and Chronic Health Evaluation II (APACHE II) scores in surviving participants were defined as secondary outcomes.

### 2.5. Statistical analysis

Descriptive statistics of patient data and clinical characteristics were compared between participants with vitamin D deficiency and those with sufficient levels using an independent samples *t* test and a Pearson Chi-square test, as appropriate. To account for repeated measurements of vitamin D, time-fixed confounders, and the longitudinal aspect of the data collected throughout the study, the primary outcome (average effect of vitamin D deficiency on 3-month GOSE) was evaluated via adjusted multilevel random-effects ordered logistic regression in the context of a mixed-effects generalized linear model. The results were reported as proportional odds ratios, estimating the odds of the highest versus the lowest GOSE scores. In addition, secondary outcomes, namely the association between baseline vitamin D deficiency and APACHE-II scores and the length of ICU stay, were evaluated using adjusted ordinary linear and logistic regression models. This analysis was carried out using Stata version 17 (StataCorp, College Station), with a significance level set at .05.

## 3. Results

### 3.1. Patient characteristics

Out of 907 enrolled cases, 300 patients met the inclusion criteria. Five hundred forty-nine patients didn’t meet the selection criteria, and 58 were lost to follow-up 3 months after admission. The mean age of patients was 34.98 ± 14.75 years, and 250 (83.3%) were male. There were 222 patients (74%) with moderate TBI and 78 patients (26%) with severe TBI. Among patients with moderate and severe brain injuries, falls (34%) were the most common cause of injury, followed by motorcycles (24.7%). After the initial evaluation of moderate and severe TBI patients, no significant correlation was found in crude analysis between vitamin D-deficient and-sufficient groups regarding sex, age, BMI, trauma mechanism, smoking, diabetes mellitus, hypertension, presence of shock, baseline GCS score, ICU length of stay, APACHE II, Simplified Acute Physiology Score III, 3-month GOSE, and 3-month mortality. However, in patients with moderate TBI, the crude analysis of Injury Severity Score and 3-month GCS revealed significant differences between the vitamin D deficiency and vitamin D sufficient groups (*P* = .031 and *P* < .001, respectively). Moreover, in moderate TBI, the rate of nosocomial infection was significantly higher in the vitamin D-deficient group (<20 ng/mL) than in the other group (*P* = .028). Detailed baseline characteristics and patient outcome data are reported in Table 1.

**Table 1:** Patients’ characteristics.

Participant data		Moderate TBI		*P*-value	Severe TBI		*P*-value*
		Vitamin D sufficient at baseline (≥20 ng/mL) n = 44	Vitamin D deficient at baseline (<20 ng/mL) n = 178		Vitamin D sufficient at baseline (≥20 ng/mL) n = 15	Vitamin D deficient at baseline (<20 ng/mL) n = 63	
Male sex (%)		36 (81.8%)	149 (83.7%)	.763	10 (66.7%)	55 (87.3%)	.054
Age (mean ± SD)		33.09 ± 14.03	35.48 ± 14.42	.323	35.80 ± 13.33	34.68 ± 16.61	.809
BMI (kg/m^2^) (mean ± SD)		24.66 ± 2.01	25.24 ± 2.14	.214	24.27 ± 2.47	25.18 ± 2.53	.214
Smoking (%)		12 (27.3%)	34 (19.1%)	.231	3 (20%)	16 (25.4%)	.662
Trauma mechanism (%)	Driver	10 (22.7%)	33 (18.5%)	.581	2 (13.3%)	14 (22.2%)	.846
	Passenger	4 (11.8%)	30 (16.9%)		2 (13.3%)	11 (17.5%)	
	Motorcycle	10 (22.7%)	47 (26.4%)		4 (26.7%)	13 (20.6%)	
	Fall	19 (43.2%)	59 (33.1%)		6 (40.0%)	18 (28.6%)	
	Slip	1 (2.3%)	6 (3.4%)		1 (6.7%)	4 (6.3%)	
	Head collision	0 (0%)	3 (1.7%)		0 (0%)	3 (4.8%)	
Diabetes mellitus (%)		17 (38.6%)	58 (32.6%)	.447	5 (33.3%)	20 (31.7%)	.906
Hypertension (%)		6 (13.6%)	32 (18.0%)	.494	5 (33.3%)	12 (19.0%)	.228
Nosocomial infection (%)		4 (9.1%)	43 (24.2%)	**.028**	3 (20%)	12 (19.0%)	.933
Presence of shock in admission (%)		5 (11.4%)	21 (11.8%)	.936	4 (26.7%)	12 (19.0%)	.511
Blunt injury (%)		36 (81.8%)	129 (72.5%)	.204	8 (53.3%)	37 (58.7%)	.704
GCS score at baseline (mean ± SD)		11.00 ± 1.08	10.95 ± 1.05	.777	5.60 ± 1.59	6.00 ± 1.87	.448
ICU length of stay		9.39 ± 9.73	9.96 ± 8.59	.702	6.73 ± 5.44	7.98 ± 8.09	.572
ISS		21.99 ± 1.48	22.72 ± 3.40	**.031**	22.06 ± 1.50	22.79 ± 3.22	.396
APACHE II		11.76 ± 1.87	12.05 ± 2.79	.524	12.79 ± 3.27	12.40 ± 3.05	.662
SAPS III		42.28 ± 2.20	42.23 ± 2.33	.913	41.71 ± 1.14	41.95 ± 2.50	.721
GCS score at 3 months (mean ± SD)		13.73 ± 2.28	11.70 ± 2.15	**<.001**	5.80 ± 2.14	5.44 ± 2.05	.551
Glasgow Outcome Score Extended (mean ± SD)		6.34 ± 1.08	6.04 ± 1.27	.157	3.20 ± 1.93	2.30 ± 1.67	.074
3-months mortality (%)		1 (2.3%)	7 (3.9%)	.597	5 (33.3%)	35 (55.6%)	.122

Bold values indicate statistically significant results.

APACHE II = Acute Physiology and Chronic Health Evaluation II, BMI = body mass index, GCS = Glasgow Coma Scale, ISS = Injury Severity Score, SAPS III = Simplified Acute Physiology Score III.

* *P*  < .05 was considered statistically significant.

### 3.2. Primary and secondary outcomes

To determine the average effect of vitamin D deficiency on a 3-month GOSE, a multilevel random-effects ordered logistic regression model was used in the context of a mixed-effects generalized linear model, adjusted for TBI severity, sex, age, smoking, diabetes mellitus, hypertension, BMI, nosocomial infection during hospitalization, and baseline GCS. The logistic regression models demonstrated that vitamin D deficiency (<20 ng/mL) was associated with an increased likelihood of poorer 3-month GOSE (proportional odds ratio [pOR] = 0.62, confidence interval: 0.40–0.93, *P* = .022, Table 2). Moreover, TBI severity, female gender (pOR = 2.37, *P* = .002), and baseline GCS (pOR = 1.46, *P* < .001) were found to be significantly correlated with the 3-month GOSE (Table 2).

**Table 2:** Assessment of vitamin D levels in relation to primary and secondary outcomes.

Predictors	Statistical measure	Estimate	95% confidence interval	*P*-value
Primary outcome* (GOSE at 3 months)				
TBI severity (severe vs moderate)	pOR	0.08	0.03 to 0.18	**<.001**
Sex (female vs male)	pOR	2.37	1.38 to 4.06	**.002**
Age	pOR	1.00	0.99 to 1.02	.549
Smoking	pOR	0.85	0.53 to 1.36	.507
Diabetes mellitus	pOR	0.80	0.52 to 1.21	.291
Hypertension	pOR	0.69	0.41 to 1.16	.166
BMI	pOR	0.96	0.88 to 1.05	.365
Nosocomial infection	pOR	0.81	0.50 to 1.32	.393
GCS at baseline	pOR	1.46	1.31 to 1.62	**<.001**
Vitamin D status (deficient vs sufficient)	pOR	0.62	0.40 to 0.93	**.022**
Secondary outcome (ICU length of stay in surviving participants)				
TBI severity (severe vs moderate)	Coefficient	−0.83	−6.08 to 4.42	.756
Sex (female vs male)	Coefficient	1.04	−1.65 to 3.73	.447
Age	Coefficient	−0.04	−0.11 to 0.03	.250
Smoking	Coefficient	−1.84	−4.35 to 0.66	.149
Diabetes mellitus	Coefficient	−1.02	−3.26 to 1.22	.372
Hypertension	Coefficient	1.48	−1.35 to 4.32	.304
BMI	Coefficient	−0.15	−0.62 to 0.31	.516
Nosocomial infection	Coefficient	2.56	−0.05 to 5.17	.055
GCS at baseline	Coefficient	0.31	−0.61 to 1.24	.508
Vitamin D status at baseline (deficient vs sufficient)	Coefficient	0.29	−2.30 to 2.87	.828
Secondary outcome (nosocomial infection in surviving participants)				
TBI severity (severe vs moderate)	OR	1.43	0.55 to 3.70	.462
Sex (female vs male)	OR	0.71	0.33 to 1.53	.381
Age	OR	1.00	0.98 to 1.03	.583
Vitamin D status at baseline (deficient vs sufficient)	OR	0.43	0.17 to 1.09	.077
Diabetes mellitus	OR	0.96	0.49 to 1.88	.913
Secondary outcome (APACHE-II score in surviving participants)				
TBI severity (severe vs moderate)	Coefficient	0.65	−1.07 to 2.37	.458
Sex (female vs male)	Coefficient	−0.17	−1.05 to 0.71	.701
Age	Coefficient	−0.02	−0.04 to 0.01	.143
Smoking	Coefficient	−0.13	−0.95 to 0.69	.757
Diabetes mellitus	Coefficient	−0.08	−0.82 to 0.65	.824
Hypertension	Coefficient	0.22	−0.71 to 1.15	.646
BMI	Coefficient	−0.07	−0.22 to 0.08	.354
Nosocomial infection	Coefficient	0.05	−0.81 to 0.90	.916
GCS at baseline	Coefficient	0.04	−0.26 to 0.35	.774
Vitamin D status (deficient vs sufficient)	Coefficient	0.53	−0.32 to 1.38	.219

APACHE-II = Acute Physiology and Chronic Health Evaluation II, BMI = body mass index, GCS = Glasgow Coma Scale, GOSE = Glasgow Outcome Scale-Extended, ICU = intensive care unit, OR = odds ratio, pOR = proportional odds ratio, TBI = traumatic brain injury.

* Adjusted effect of vitamin D deficiency averaged over repeated measurements with random effects added per subject.

*P* < .05 was considered statistically significant and is indicated in bold.

Multivariate analyses adjusted for TBI severity, sex, age, smoking, diabetes mellitus, hypertension, BMI, nosocomial infection during hospitalization, and baseline GCS did not reveal any association between ICU length of stay (odds ratio [OR]: 0.29, *P* = .828), nosocomial infection (OR: 0.43, *P* = .077), and APACHE II scores (OR: 0.53, *P* = .219) in surviving participants (Table 2).

## 4. Discussion

In this study, the primary evaluation of TBI patients at a 3-month follow-up revealed that vitamin D status was not significantly associated with most baseline characteristics or early clinical outcomes, including APACHE II scores, ICU length of stay, or the incidence of nosocomial infection. Consistent with our findings, several previous studies have also reported no meaningful correlation between vitamin D levels and factors such as age, gender,^[25–27]^ BMI,^[28]^ ICU length of stay,^[29]^ mechanism of trauma, diabetes, hypertension,^[25,26]^ smoking status, or initial GCS^[25,27,30]^ in individuals with TBI. However, to the best of our knowledge, no studies have specifically examined vitamin D status in relation to nosocomial infections or APACHE II scores exclusively in TBI patients, though these outcomes have been investigated in broader critical care populations with conflicting results.^[31–35]^ In this context, some studies have found that low vitamin D levels correlate with higher APACHE II scores, suggesting that vitamin D deficiency may reflect greater illness severity due to its role in immune function, metabolic regulation, and systemic inflammatory responses.^[31,36]^ These findings imply that vitamin D status could serve as a prognostic biomarker in critically ill patients. Conversely, other studies did not observe a significant correlation between vitamin D levels and APACHE II scores in critically ill patients,^[32,33]^ which is consistent with our results. These discrepancies may arise from differences in sample sizes, the timing of vitamin D measurement relative to illness onset, and varying thresholds used to define deficiency, all of which affect the sensitivity of detecting true relationships. Similarly, the link between vitamin D deficiency and nosocomial infections is debated. Vitamin D is known to modulate both innate and adaptive immunity, and deficiency has been hypothesized to increase the risk of infection by impairing immune defenses.^[34,37,38]^ Some investigations support this hypothesis, reporting higher rates of hospital-acquired infections in patients with vitamin D deficiency.^[39,40]^ However, other studies in general critical care populations,^[33,41,42]^ as well as our adjusted TBI-specific analysis, found no statistically significant association between lower vitamin D levels and nosocomial infection, though our data showed a marginal trend (*P* = .077). These conflicting results may stem from heterogeneity in patient populations, differences in infection surveillance and definitions, and challenges in accurately assessing vitamin D status in critically ill patients. In this regard, factors such as timing of serum 25(OH)D measurements, small effect sizes, the absence of direct vitamin D storage measurements, lack of a true placebo, and genetic differences in vitamin D action have been identified as some of the existing challenges.^[43]^ Understanding these nuances is essential for interpreting our findings and highlights the need for larger, standardized studies to clarify the prognostic value of vitamin D in this context.

In the present study, the role of vitamin D on 3-month mortality and GOSE outcome was investigated. Our results demonstrated that vitamin D deficiency (defined as a level of < 20 ng/mL), assessed longitudinally with measurements on admission, 7 days, and 3 months post-admission was associated with an increased likelihood of poorer GOSE. Nevertheless, as with other studies,^[12,25,44]^ our findings did not reveal an association between baseline vitamin D levels and 3-month mortality. Conversely, some studies have revealed that vitamin D deficiency is significantly associated with higher in-hospital mortality^[45]^ and poorer 3-month post-discharge Glasgow Outcome Scale scores^[46]^ in patients admitted to a neurocritical care unit. However, Karsy et al^[16]^ conducted a double-blind, placebo-controlled, randomized (1:1) clinical trial involving 274 patients with neurocritical care who had vitamin D deficiency. Consistent with our results, they found no significant differences in hospital length of stay, ICU length of stay, or 30-day mortality. Therefore, they concluded that although some studies suggest that vitamin D may predict prognosis, supplementation with vitamin D in vitamin D-deficient neurocritical care patients did not lead to meaningful improvements in outcomes and is unlikely to influence acute clinical recovery.^[16]^

To date, the role of vitamin D in some outcomes of brain injury patients has been investigated. In this regard, a retrospective study involving 345 patients with TBI evaluated the effect of serum Vitamin D levels on performance function (GOSE) and cognitive outcomes. The study revealed that vitamin D supplementation during the acute phase of a mild-to-moderate TBI may improve both performance and cognitive outcome over the long term.^[25]^ However, although the retrospective nature of that study and the type of patient selection make direct comparison with our work difficult, previous research suffers from a lack of multivariate analysis. In the present study, the adjusted multilevel regression analysis revealed a significant association between vitamin D deficiency and poorer GOSE scores (*P* = .022). Consistent with our results, Sharma et al found that in 35 patients with moderate to severe brain injury admitted to the ICU, vitamin D supplementation was associated with a higher GOSE score after 14 days.^[12]^ A significant proportion of what is known about vitamin D’s neuroprotective effects stems from data on vitamin D deficiency, which suggests that it regulates apoptosis and reduces inflammation, oxidative stress, and excitotoxicity.^[47]^ In the context of TBI, vitamin D has also been studied alongside progesterone to explore their potential synergistic effects and to examine the relationship between age-related vitamin D decline and brain injury.^[48]^ Although the combination of vitamin D and progesterone proved effective in adult rats, it appears to offer the greatest benefits in middle-aged animals, possibly due to their greater vitamin D deficiency. Moreover, this combination has been shown to significantly reduce astrocyte proliferation and neuronal loss in older animals.^[49]^ Additionally, other studies have indicated that this combination improves cognitive function and reduces inflammation and neuron loss.^[50,51]^ It has not been fully elucidated why vitamin D and progesterone synergize. However, a study suggests that this may be due to a decrease in astrocyte activation and the phosphorylation of nuclear factor kappa-light-chain-enhancer of activated B cells.^[51]^ According to Yang et al, vitamin D supplements decrease brain edema and inflammation while enhancing blood–brain barrier integrity and behavioral function following TBI.^[52]^

Additionally, a 2020 study involving 35 patients with moderate TBI found that those randomly assigned to receive one-time doses of vitamin D supplementation demonstrated improved consciousness at 7 days and better GOSE at 14 days compared to controls,^[12]^ confirming previous findings.^[27]^ In this regard, similar results were also observed in our investigation. Overall, since vitamin D supplements are inexpensive and generally safe, their use may be beneficial in improving the clinical outcomes of TBI patients by reducing inflammation and infection-related mortality.^[53]^

### 4.1. Limitations

The present study had limitations. Due to the complex and partially understood nature of the pathophysiology of TBI and the differences in post-discharge care, self-care, and nutritional status of these patients, evaluating the effect of vitamin D on central nervous system function posed significant challenges. Importantly, since withholding treatment from vitamin D-deficient patients was deemed unethical, all such individuals without contraindications received supplementation during the first week of admission. This may have attenuated the observed association between deficiency and clinical outcomes. However, repeated measurements of Vitamin D status and investigating the association via average effects likely helped mitigate confounding. Additionally, the single-center design may limit the generalizability of our findings. It is also worth noting that research on the role of vitamin D supplementation in TBI is still in its early stages. Given the ongoing uncertainties in the literature regarding the optimal dosage, formulation, mechanisms of action, and therapeutic role of vitamin D in TBI recovery, the interpretation of our findings should remain cautious. Further large-scale randomized controlled trials are recommended to clarify optimal timing, form, and dosage of vitamin D supplementation, as well as its efficacy in this patient population.

## 5. Conclusion

The results of this study suggest that vitamin D deficiency is associated with poorer neurological outcomes, as assessed by the GOSE, at 3 months. The potential role of vitamin D status as a prognostic marker and the effect of its supplementation in TBI patients should be investigated in future large-scale randomized controlled studies.

## Author contributions

**Conceptualization:** Mehdi Mahmoodkhani, Arvin Rahimi, Arvin Naeimi.

**Data curation:** Arvin Rahimi, Arvin Naeimi, Sepehr Aghajanian.

**Formal analysis:** Arvin Naeimi, Sepehr Aghajanian.

**Investigation:** Mehdi Mahmoodkhani, Arvin Rahimi, Arvin Naeimi.

**Methodology:** Mehdi Mahmoodkhani, Arvin Rahimi, Arvin Naeimi.

**Project administration:** Mehdi Mahmoodkhani, Arvin Rahimi.

**Resources:** Mehdi Mahmoodkhani.

**Software:** Arvin Naeimi, Sepehr Aghajanian.

**Supervision:** Mehdi Mahmoodkhani.

**Validation:** Mehdi Mahmoodkhani, Arvin Naeimi.

**Visualization:** Arvin Naeimi, Sepehr Aghajanian.

**Writing – original draft:** Mehdi Mahmoodkhani, Arvin Rahimi, Arvin Naeimi, Sepehr Aghajanian, Anita Naeimi.

**Writing – review & editing:** Arvin Naeimi, Anita Naeimi.
